# A conceptual model to guide research on the activities and effects of innovation champions

**DOI:** 10.1177/2633489521990443

**Published:** 2021-03-23

**Authors:** Christopher M Shea

**Affiliations:** Gillings School of Global Public Health, The University of North Carolina at Chapel Hill, Chapel Hill, NC, USA

**Keywords:** Champion, innovation, implementation strategy, organizational change

## Abstract

**Background:**

The importance of having a champion to promote implementation efforts has been discussed in the literature for more than five decades. However, the empirical literature on champions remains underdeveloped. As a result, health organizations commonly use champions in their implementation efforts without the benefit of evidence to guide decisions about how to identify, prepare, and evaluate their champions. The goal of this article is to present a model of champion impact that draws upon previous literature and is intended to inform future research on champions and serve as a guide for practitioners serving in a champion role.

**Methods:**

The proposed model is informed by existing literature, both conceptual and empirical. Prior studies and reviews of the literature have faced challenges in terms of operationalizing and reporting on champion characteristics, activities, and impacts. The proposed model addresses this challenge by delineating these constructs, which allows for consolidation of factors previously discussed about champions as well as new hypothesized relationships between constructs.

**Results:**

The model proposes that a combination of champion commitment and champion experience and self-efficacy influence champion performance, which influences peer engagement with the champion, which ultimately influences the champion’s impact. Two additional constructs have indirect effects on champion impact. Champion beliefs about the innovation and organizational support for the champion affect champion commitment.

**Conclusion:**

The proposed model is intended to support prospective studies of champions by hypothesizing relationships between constructs identified in the champion literature, specifically relationships between modifiable factors that influence a champion’s potential impact. Over time, the model should be modified, as appropriate, based on new findings from champion-related research.

## Plain language summary

An innovation champion is an individual who works within an organization and who dedicates themselves to promoting a change within the organization, such as implementing a new intervention or a new quality improvement effort. Health organizations commonly rely on innovation champions, and existing literature on champions suggests they are important for successful organizational change. However, many questions remain about what effective champions do and what types of support they need to perform their champion role well. The goal of this article is to present a model of champion impact that draws upon previous literature and is intended to serve as a guide for future research on champions. In doing so, the model could support coordinated research efforts that answer questions about the characteristics, activities, and impacts of champions. Ultimately, this research could lead to development of useful guidance and tools for health system leaders to support champions within their organizations.

A champion is an individual who is “the face” of an implementation effort—one “who dedicate[s] themselves to supporting, marketing, and driving through an implementation, overcoming indifference or resistance that the intervention may provoke in an organization” (Powell et al., 2015). Champions are commonly employed in health care when implementing new interventions and undertaking quality improvement efforts, and a recent systematic review indicates that champions also are the subject of increasing interest among researchers (Miech et al., 2018). Champions have been studied in several health service settings, such as primary care (Bentz et al., 2007; Papadakis et al., 2014), hospitals (Acolet et al., 2011; Damschroder et al., 2009; Ellerbeck et al., 2006), and long-term care facilities (McCabe et al., 2013), and these studies have focused on various interventions, such as tobacco cessation treatment (Bentz et al., 2007; Papadakis et al., 2014), mental health integration (Chang et al., 2013), weight management (Kahwati et al., 2011), and immunizations (Albert et al., 2012; Tierney et al., 2003). Champions also have been examined in the context of various health information technologies (IT), such as electronic health records, provider order-entry systems, and telehealth (Ash et al., 2003; Paré et al., 2011; Shea et al., 2016; Taylor et al., 2015).

The existing literature on champions has yielded some interesting, though descriptive, findings. In general, evidence suggests that champions contribute to successful implementation (Miech et al., 2018); however, there are exceptions (Acolet et al., 2011; Markham, 1998; McCabe et al., 2013; Naylor et al., 2006). Notably, most available research has treated champions as a dichotomous variable (i.e., presence or absence of a champion; Miech et al., 2018; Shea & Belden, 2016), which does not account for the many ways that champions may differ. For example, studies have found that champions hold various roles in their organizations (e.g., clinical, middle management, IT, senior leadership; Day, 1994; Maidique, 1980; Miech et al., 2018), and some champions represent multiple roles (e.g., clinical and IT), enabling them to serve as boundary spanners across organizational units (Ash et al., 2003; Clack et al., 2018). Studies also have noted the presence of multiple champions within a single implementation effort (Damschroder et al., 2009; Shaw et al., 2012) and champions from different organizations working together for a common purpose (Gupta et al., 2006). Despite useful descriptive findings such as these, a clear gap exists in understanding what makes a champion effective, specifically in health services organizations. According to Meich et al.’s review, “Few studies have attempted to isolate and measure a specific ‘champion effect,’ or to describe and explain the particular mechanisms by which champions influence implementation processes and related outcomes” (Miech et al., 2018). This overall assessment suggests that the state of champion research has not advanced significantly since the early 2000s, when a systematic review by Greenhalgh et al. indicated that there is “remarkably little direct empirical evidence on how to identify, and systematically harness the energy of, organisational champions” (Greenhalgh et al., 2004).

Important for advancing knowledge on what makes a champion effective are studies of well-specified champion strategies (Proctor et al., 2013). The purpose of this article is to propose a model that is grounded in existing literature, both conceptual and empirical, to inform such studies. These studies should lead to evidence-based answers to several questions important to practitioners, including the following: Which characteristics and experiences are important when selecting champions? Which activities should champions perform, and which implementation outcomes can they affect? What types of organizational support do champions need to perform the champion role effectively? How do champion activities and outcomes change during the course of an implementation effort? Ultimately, future findings could facilitate development of guidance and tools to support selection and preparation of champions. The proposed model could also be a useful guide for practitioners serving in a champion role, particularly those with little or no experience in such a role.

## Methods

### Literature review

Development of the proposed model was guided by existing literature on champions. Given the recent integrative review on champions in health care (Miech et al., 2018) and systematic review on clinical champions in substance use and mental health (Wood et al., 2020), a new systematic review did not seem warranted. Instead, the model development drew upon the work of these two reviews, relevant studies published after these reviews, and foundational work on champions published in non-health-related sources. The champion concept has been discussed in the management (Schon, 1963), technology, (Chakrabarti, 1974; Howell & Higgins, 1990a), and innovation (Rogers, 2003) literatures for decades. More recently, the concept has been included in quality improvement methods, such as Six Sigma, (Snee, 2001), and prominent implementation frameworks, such as the Consolidated Framework for Implementation Research (Damschroder et al., 2009).

### Methods Champions as an implementation strategy

In the implementation science literature, champions have been identified in compilations of strategies (Leeman et al., 2007; Powell et al., 2012). In the compilation from the Expert Recommendations for Implementing Change (ERIC) project, the champion strategy is labeled as “identifying and preparing champions” (Powell et al., 2015), which is notable for a couple reasons. First, champions historically were thought of as being emergent—individuals who assumed the role of champion for a cause they believed in (Howell and Higgins, 1990b; Schon, 1963). However, in current practice, many organizations appoint individuals to champion roles as an implementation strategy (Wood et al., 2020). This distinction between emergent and appointed champions has been recognized in the literature (Damschroder et al., 2009; Soo et al., 2009); however, we do not have much evidence about differential effects of emergent and appointed champions, let alone why one type may be more effective than the other. Given an apparent trend toward appointing champions and potentially insufficient training being provided for the champion role (Wood et al., 2020), identifying approaches for selecting, preparing, and supporting effective champions should be a priority for the field. In fact, expert consensus suggests that identifying and preparing champions is one of the more important and feasible implementation strategies (Waltz et al., 2015).

Labeling the strategy as “identifying and preparing champions” is also important because it suggests that the strategy is employed not by the champion but instead by some other organizational member, presumably one holding a leadership position. However, the full range of responsibilities performed by the champion is not specified in the compilation of strategies and remains a gap in the field, as highlighted by a recent study (Goedken et al., 2019). In implementation efforts that use multiple strategies to address different levels of barriers (Weiner et al., 2012), the strategy of identifying and preparing champions likely would be employed within a multifaceted implementation approach, involving other discrete strategies, some of which the champions themselves would lead or be involved with employing. Within the ERIC compilation, the strategy of identifying and preparing champions has been categorized within a group of strategies focused on developing relationships between stakeholders; examples of other strategies included in this group are “recruit, designate, and train for leadership” and “inform local opinion leaders” (Waltz et al., 2015). Although this categorization suggests that champions may focus on developing stakeholder relationships, we should not assume all strategies within the category would be employed by champions. Just as “identifying and preparing champions” presumably would be performed by a member of the organization’s leadership, so would “recruit, designate, and train for leadership.” However, a champion may be involved with “informing local opinion leaders.” In summary, a gap remains in the literature regarding which strategies champions should employ and when, as well as what distinguishes champions who effectively employ these strategies from those champions who do not.

### Champions, opinion leaders, and professional roles

Although various types of champions appear within the literature, such as “innovation champion” (Rogers, 2003) and “product champion,” (Schon, 1963) definitions of these champions generally share common elements. Such elements include being an organizational member (not an external agent) and being dedicated to achieving success of the effort, often demonstrated by bridging intra-organizational boundaries and overcoming inertia and resistance to change (Damschroder et al., 2009; Miech et al., 2018; Powell et al., 2015; Rogers, 2003). However, when researchers conflate the champion concept with concepts that may appear similar but have notable differences in their definition, ambiguity arises about what a champion is, what a champion does, and how to aggregate results of champion studies within literature reviews (Flodgren et al., 2011; Grimshaw et al., 2006; Locock et al., 2001). One example of such a concept is “opinion leader,” which is an individual with the ability to influence the beliefs of other individuals, generally about multiple topics or issues (Rogers, 2003). A champion, however, takes an active role in implementing a new intervention or change effort, during which they aim to influence beliefs specifically about that particular intervention or change effort (Curley & Gremillion, 1983; Damschroder et al., 2009). Although an opinion leader could serve in a champion role (Greiver et al., 2011), opinion leaders are not necessarily champions, and vice versa (Grimshaw et al., 2006).

Also important is recognizing differences between a formal professional role (e.g., clinician, administrator) and a champion role for a given implementation effort. Birken et al.’s (2012) systematic review on middle managers illustrates this point as it identifies several champion-like activities that middle managers can perform such as “diffusing information” and “selling innovation implementation.” Clearly, a middle manager may perform a champion role. However, we cannot assume that all middle managers are champions and that all champions are middle managers—just as we cannot assume that all opinion leaders are champions and all champions are opinion leaders. In summary, a champion may hold one of many types of roles within an organization (e.g., physician, nurse, administrator), actively promotes and participates in leading a specific implementation or change initiative, serves as a bridge between stakeholder groups, and may (or may not) be formally appointed by leadership to do so.

### Champion characteristics and activities

Notably, studies have identified various types of champion characteristics (e.g., personality attributes, knowledge) and activities (e.g., advocating for the innovation to leadership). However, differentiating between characteristics and activities has proved problematic, contributing to gaps in theory and measurement, and, ultimately, hindering efforts to identify which activities effective champions perform and which characteristics champions need to perform these activities effectively. This challenge is illustrated by Howell and Higgins’s foundational theory on champion emergence, which includes personality characteristics (e.g., risk taking, innovation, social adroitness), transformational leadership behaviors (e.g., charisma, inspiration, intellectual stimulation), and influence tactics (e.g., building coalitions, appealing to higher authority, bargaining; Howell & Higgins, 1990b). For example, is “risk taking” clearly a characteristic and not a behavior? Is “charisma” a behavior or a characteristic? The review by Meich et al. also illustrates this challenge. For example, the review discusses “communication across organizational boundaries” as a characteristic, although communication could be considered an activity; “serving as a team leader” as a characteristic, with “leading teams and recruiting new team members” as an activity; and “engaging in planning activities” as an activity, with “engaging in team planning and goal-setting” as a characteristic (Miech et al., 2018).

To help address this challenge, the proposed model aims to distinguish between types of *characteristics* (i.e., beliefs or attributes of an individual) and *activities* (i.e., tasks that an individual performs), while acknowledging that characteristics may influence the performance of activities. For example, demonstrating effective use of the innovation presumably requires both characteristics (e.g., knowledge about and experience with the innovation) and activities (e.g., role modeling, answering questions about how to use the innovation). Without the necessary characteristics, a champion may not perform the activities effectively. Specifying characteristics and activities should facilitate better measurement of the champion construct—beyond the dichotomous presence or absence of a champion.

## Results

### The proposed model

**Figure 1.**
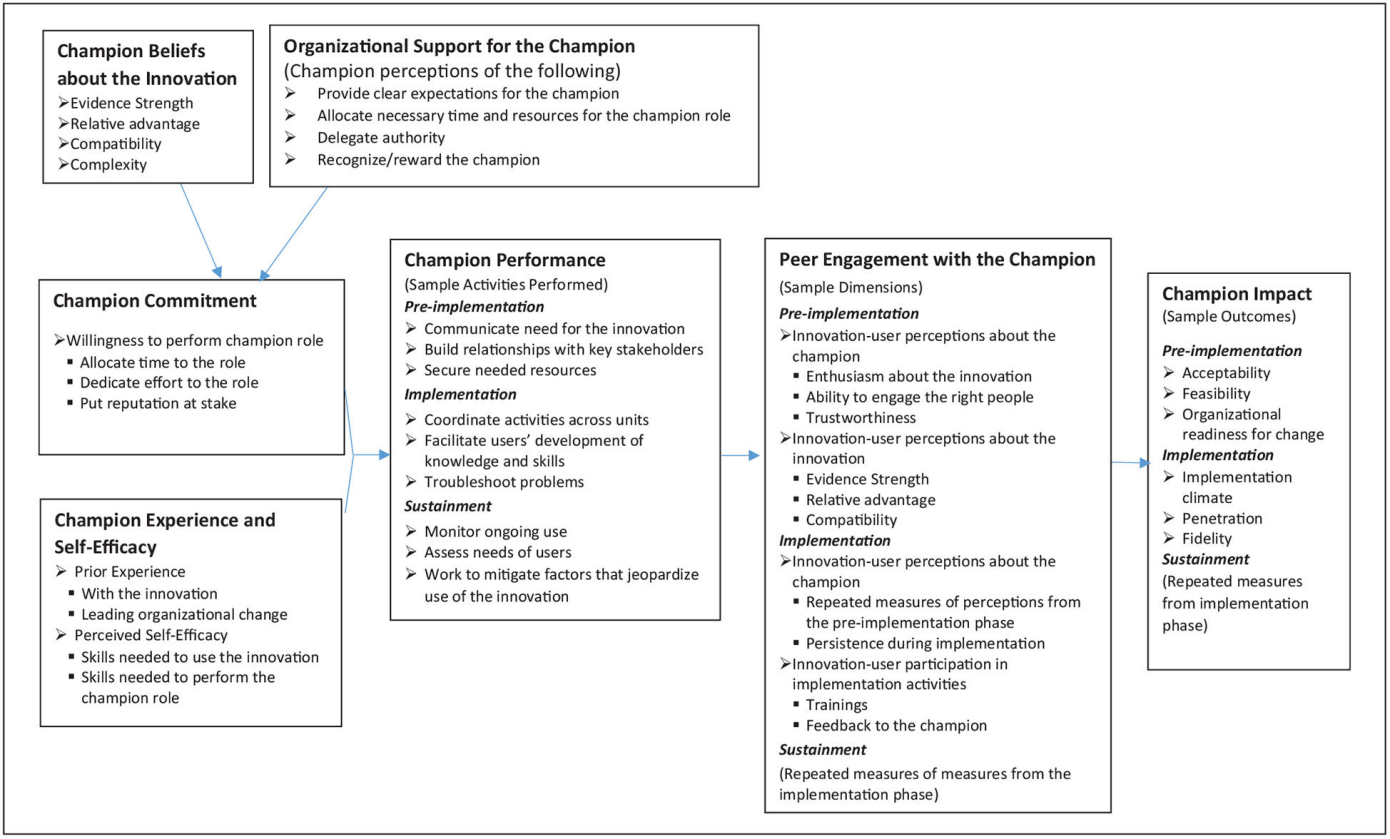
A conceptual model of champion impact with selected dimensions.

The proposed model consists of seven constructs. Broadly speaking, the model suggests that the combination of *champion commitment* and *champion experience and self-efficacy* influence *champion performance*, which influences *peer engagement with the champion*, which ultimately influences the *champion impact*. The remaining two constructs have indirect effects on *champion impact. Champion beliefs about the innovation* and *organizational support for the champion* affect *champion commitment*. (See Figure 1 for a condensed version of the model. Supplemental Figure 1 includes additional dimensions of the constructs to consider. It is not feasible to include all possible dimensions in the figure, so researchers are encouraged to examine other dimensions of interest to operationalize the model constructs.) Notably, there is not one single construct of “champion characteristics.” Differentiating between types of characteristics is important because their effects may vary across innovations and settings (Rogers, 2003). Furthermore, personality characteristics, which have been commonly cited in prior studies, are not included in the model, for a few reasons. First, as noted above, prior work has illustrated the challenge of clearly differentiating (i.e., operationalizing) characteristics, behaviors, and activities. Second, the effect of personality characteristics on champion impact likely occurs through their influence on champion activities. Finally, personality characteristics tend not to be easily modifiable. The model focuses on modifiable beliefs, attributes (e.g., knowledge, skills, experience), and activities, which is useful for examining how to prepare and “systematically harness the energy of” champions (Greenhalgh et al., 2004).

The constructs *champion performance* and *champion impacts* reflect the dynamic nature of champion efforts (Damsgaard & Scheepers, 2000; Grazioli et al., 2019; Howell & Boies, 2004), suggesting activities and impacts differ across pre-implementation (e.g., planning), implementation (e.g., executing), and sustainment phases (e.g., ongoing evaluation and improvement; Chamberlain et al., 2011). Clearly, implementation processes and quality improvement methods are not always linear (Aarons et al., 2011); however, even iterative approaches may involve completing phases (e.g., pre-work, active implementation, and sustainment) within a cyclical fashion. Notably, the specific characteristics, activities, and outcomes included in the proposed model are not intended to be a comprehensive list or recipe for all studies. The model aims to identify key domains and propose relationships between them as a basis for future research; however, researchers are encouraged to select specific variables and outcomes that are appropriate for their study.

### Results Champion commitment

*Champion commitment* involves the individual’s willingness to perform the champion role. This construct encompasses dimensions that have been identified in the literature previously, specifically a willingness to “go the extra mile” (Rycroft-Malone et al., 2019) by dedicating time and energy (Howell et al., 2005) to the implementation effort and, potentially, risking one’s reputation (e.g., if the implementation fails; Beath, 1991). More recently, studies have found that successful champions demonstrated motivation and commitment to the implementation by spending substantial time and effort on implementation activities (Bonawitz et al., 2020; Bunce et al., 2020). These studies support the proposed, positive relationship in the model between champion commitment and champion performance of activities that require a substantial investment of time and effort. Furthermore, recognizing the multi-dimensionality of champion commitment is important because appointed and emergent champions may not have the same level of commitment across these dimensions. In fact, the study by Bonawitz suggests that appointed champions in their sample did not have the level of commitment necessary to inspire successful change (Bonawitz et al., 2020). Although supported in that study sample, we should not assume that appointed champions cannot demonstrate commitment to the role. In fact, even the idea that *appointed and emergent* are clearly dichotomous is an open question. For example, a champion may begin to emerge before being formally appointed, with the emergence being a contributing factor in the organizational leadership’s decision to appoint the champion. A key point here is that leaders who appoint a champion want the champion to take ownership of the idea (Bonawitz et al., 2020), just as an emergent champion would (Schon, 1963). Therefore, the model proposes that the champion’s commitment is directly influenced by the *champion’s beliefs about the innovation*. In other words, even appointed champions could demonstrate commitment if they believe the innovation is a “good idea.” Such a belief is consistent with seminal frameworks—Rogers’ (2003) Diffusion of Innovations and Damschroder et al.’s (2009) CFIR—for example, beliefs about evidence supporting the innovation, compatibility, complexity, and relative advantage compared to the status quo. According to the proposed model, however, such beliefs are not the only proposed determinant of champion commitment. In fact, for some champions, it is possible that other factors (discussed below) are more influential than their beliefs about the innovation. The proposed model aims to inform studies to test such hypotheses.

For a champion to be committed, in addition to thinking that the innovation is a good idea, they likely will want to believe that their organization will support them. Therefore, consistent with social exchange theory, the model proposes that the champion will feel more committed to their role if they have the organization’s support (Eisenberger et al., 1990). Prior studies have identified challenges that champions face, such as having time to complete both day-to-day operational activities and champion activities (Bonawitz et al., 2020), which could be addressed with organizational support. Aspects of organizational support have been included in only a small number of champion studies, such as providing clear expectations about what the champion role involves and time for staff to support the champion in their efforts (Beath, 1991); training to perform the champion role (Hagedorn et al., 2019; Helseth et al., 2018); requisite decision-making authority (Howell & Higgins, 1990a); and recognition and rewards, such as financial incentives or pay increases and promotion (Eisenberger et al., 1990; Howell & Higgins, 1990a). Beath (1991) points out that information technology champions commonly rely on consultants, particularly if inhouse staff time is not available. For champions with little or no experience leading implementation efforts, similar support could come from an implementation science consultant to coach the champion, particularly if an individual within the organization is not available to mentor the champion. More recently, Bunce and colleagues (2020) found that “implementation success depended on both the presence of champions with the aforementioned attributes and the implicit or explicit backing of clinic leadership, and the interaction of the two.” The champion attributes they refer to include “interest in and willingness to promote the intervention,” “sufficient social capital to foster trust and the authority to prioritize implementation and stimulate practice change,” “creditability conferred through prescribing privileges,” and “time—and understanding of [the intervention] sufficient to effectively advocate for the intervention” (Bunce et al., 2020). The authors “defined organizational support as the creation of an environment within which implementation activities could be expected to be taken seriously by clinic staff” and found that organizational support took “many forms,” including selecting a champion with the attributes listed above, including an individual with available time for the role, and prioritizing the intervention even as the organization pursues other initiatives (Bunce et al., 2020). These findings, as well as findings in studies focused on complementary topics such as organizational readiness, climate, and implementation effectiveness (Helfrich et al., 2007, 2009; Jacobs et al., 2014) suggest that organizational support for the champion requires more attention in future research. Even capable champions likely will struggle to perform their day-to-day organizational role in addition to their champion role under unsupportive conditions.

### Champion experience and self-efficacy

The model proposes that being committed to the champion role is not sufficient. A champion must also have relevant knowledge, skills, and experience. Therefore, *champion experience and self-efficacy*, combined with *champion commitment*, are hypothesized to influence champion performance. *Champion experience and self-efficacy* include (1) experience with both the innovation and with leading organizational change and (2) perceived self-efficacy with both using the innovation effectively and performing the champion role effectively. Experience with the innovation is important because it reflects a richer understanding of how to use it and challenges that may be encountered with its use (Aarons et al., 2014). Experience leading organizational change is important because implementation is a collective effort. Transformational leadership behaviors, which have been widely studied, focus on a leader’s ability to communicate the need for a change and inspire others to pursue the change (Farahnak et al., 2020). In addition to positively correlating with the implementation behaviors of organizational members (e.g., those working under the transformational leader; Michaelis et al., 2010), transformational leadership behaviors have been found to be more common in champions than in non-champions (Howell & Higgins, 1990b). In addition to transformational leadership behaviors, prior experience leading organizational change, specifically within the organization, could also influence their ability to perform such activities as building relationships with key stakeholders and troubleshooting problems that arise during implementation. Despite their importance, however, recent evidence suggests that a champion’s behaviors alone are not enough to explain implementation success (Bonawitz et al., 2020), which is why the proposed model highlights the effect of both *champion commitment* and *champion experience and self-efficacy* on *champion performance*.

### Champion performance

The proposed model identifies and categorizes several champion activities within *champion performance* (Howell et al., 2005; Miech et al., 2018; Shea & Belden, 2016). These activities are consistent with implementation strategies previously documented, for example, in the ERIC compilation of strategies (Powell et al., 2012, 2015) Activities within the pre-implementation stage include communicating the need for and benefits of the innovation, building relationships with key stakeholders, developing an implementation plan, and securing needed resources (Howell & Shea, 2006). The champion may use multiple venues and methods of communication (e.g., face-to-face, electronic) to convey what the innovation is and why it is important (Mørk et al., 2018). Also, champion communication may reinforce, or be reinforced by, communication from the organization’s leadership about the importance of the innovation. Regarding relationships with key stakeholders, champions may not have existing relationships in place and, therefore, may need to develop new relationships (Ash et al., 2003; Howell et al., 2005; Rogers, 2003). Regardless of whether the relationships are pre-existing or not, having strong inter-personal relationships is important for addressing resistance to the implementation or change effort (Damschroder et al., 2009).

During the implementation phases, a champion may perform a coordinator role, aligning activities across organizational units (Howell & Shea, 2006) and serving as a conduit for information about the innovation and the implementation (Miech et al., 2018). In addition, champions facilitate development of knowledge and skills needed for effective use of the innovation, for example, by role modeling (Gordon et al., 2011), providing one-on-one mentoring (Wood et al., 2020), or organizing tailored training (Ash et al., 2003). Champions also monitor use of the innovation (Goedken et al., 2019) and trouble-shoot various problems that arise during implementation (Howell et al., 2005). Champions continue their work in the sustainment phase (Shelton et al., 2018), for example, by continuing to monitor use of the innovation and investigating changes in patterns of use (Laur et al., 2018). They also scan the external environment to identify opportunities and assess the needs of intervention users (clinicians and/or patients; Howell & Shea, 2006).

It is important to recognize that simply executing an activity may not be enough to yield the desired effect. How well the activity is performed likely influences the impact on desired implementation outcomes, such as adoption, penetration, and fidelity (Proctor et al., 2011). Therefore, relying solely on dichotomous measures of whether a champion activity was performed is not optimal. Consistent with calls for greater specificity of implementation strategies, capturing dimensions of performance of the activity (e.g., specific actions, temporality, and dose) is preferable (Proctor et al., 2013). Data on champion activities may come from various sources, for example, direct observation, activity logs, (Bunger et al., 2017), and administrative documentation (e.g., meeting minutes). Finally, the perceptions of innovation-users about the activities that the champions perform are important for assessing how well the activities have been performed. These perceptions are captured within the construct of *peer engagement with the champion*.

### Peer engagement with the champion

The construct *peer engagement with the champion* mediates the relationship between *champion performance* and *champion impacts* because successful implementation does not occur through the effort of a champion alone. Other organizational members need to buy-in to the vision and follow the champion’s lead in participating in the change effort, which (as described above) is why prior work has connected the champion role with transformational leadership (Howell & Higgins, 1990b). Engaging in the change effort requires not only buy-in with the vision but also identification with the champion (Hater & Bass, 1988). Peers may judge champions based on their reputation (Heng et al., 1999; Lawless & Price, 1992), with more effective champions being well respected (Saint et al., 2008). Therefore, the model proposes that the pre-implementation and implementation phases of engagement include peer perceptions of the champion, specifically their trustworthiness, reliability, enthusiasm about the innovation and its implementation, and persistence during implementation (Howell et al., 2005). The model also includes two concepts, trustworthiness and reliability, which have not been a focus in the champions literature, but have been explored in the literature on relationship quality within organizations (Dorsch et al., 1998). Future research should assess whether perceptions of these attributes are indeed influential.

In the pre-implementation phase, innovation-user perceptions of the innovation itself are also key measures of engagement and, therefore, predictors of champion impact. The same concepts should be used for innovation-user perceptions as are used for the champion’s perceptions of the innovation, for example, evidence strength, feasibility, compatibility, complexity (Damschroder et al., 2009; Rogers, 2003). In the implementation phase, innovation-user participation in implementation activities (e.g., trainings; providing consistent, high-quality feedback to the champion) are also important.

### Champion impact

Similar to *champion performance*, the model suggests *champion impacts* on implementation outcomes vary across phases of implementation. *Champion performance* may directly influence appropriate implementation outcomes for each phase (Proctor et al., 2011) or indirectly influence these implementation outcomes through their determinants, such as organizational readiness for change (Weiner, 2009) and implementation climate (Weiner et al., 2011). The model proposes that effective performance of pre-implementation activities (e.g., developing implementation policies/practices, securing necessary resources) promotes positive beliefs about the value of the innovation and, ultimately, collective readiness for its implementation. Outcomes of these activities include increased acceptability, appropriateness, and feasibility of the innovation (Proctor et al., 2011).

During the implementation stage, the champion continues to work toward a supportive implementation climate in which targeted users perceive use of the innovation to be expected, supported, and rewarded (Jacobs et al., 2014; Weiner et al., 2011). Of course, champion’s work toward this aim begins pre-implementation; however, the policies and practices that shape the intervention-users’ perceptions of implementation climate may continue beyond the pre-implementation phase and such perceptions may not fully form until use of the innovation begins (Weiner et al., 2011). In addition to developing a supportive implementation climate, two relevant implementation outcomes at this stage include penetration and fidelity, as the champion aims to engage all targeted users in effective use of the innovation (Proctor et al., 2011).

In the sustainment stage, champions should continue to monitor use of the innovation and mitigate the effects of external factors that threaten sustainability, such as new reporting requirements and implementation of additional services, which could change individual beliefs about the need for (or feasibility of) continued use of the innovation and/or divert resources away from it (Howell & Shea, 2006). Assuming, of course, that the innovation is still desirable from an outcomes perspective and not a candidate for de-implementation (Prasad & Ioannidis, 2014), potential outcomes for the sustainment phase include longitudinal measures of penetration, fidelity, and cost (Proctor et al., 2011).

## Summary and future directions

The goal of the proposed model is to promote research on how to identify and prepare champions and how champion performance affects implementation outcomes. The model could also serve as a useful guide for practitioners serving in a champion role. The model is intended to be applicable for both emergent and appointed champions, holding any organizational role (e.g., senior leadership, clinician), and implementing any type of innovation in a clinical setting. The general nature of the model allows for testing hypotheses about differences across these aspects, for example, whether some types of organizational support are more important for champions in a specific organizational role (e.g., physician, nurse) or whether peer engagement with the champion is more influential for specific types of innovations.

Another goal of the model is to inform measure development for assessing champion characteristics, performance, and impacts. Many studies have measured only presence or absence of a champion, which clearly is not sufficient. Although a small number of champion-related measures exist in the literature (e.g., Helfrich et al., 2009; Howell et al., 2005), they do not capture the multidimensional aspects of champion characteristics, performance, organizational support, and impacts—a point supported by a recent review of instruments, which identifies “engaging champions” as a construct lacking valid measures (Lewis et al., 2015). The model could be used to guide selection and adaptation of existing survey items and development of new items. Fortunately, there are existing measures, many of which were not developed specifically for champion studies, that could be used for several constructs in the model (see Table 1). Although some measure development likely will be needed, including non-survey measures of champion performance (e.g., report templates, activity logs), there likely is not a need to develop a new, lengthy survey of champion impact, for a couple of reasons. First, the full range of champion performance measures and champion impact measures would not need to be collected at the same point in time. Instead, measures collected should be only those pertinent to the given implementation phase. Of course, repeated measures of some constructs would be ideal, particularly to study champion activities and impacts in the sustainment phase. Second, it would be advisable to use measures for multiple aspects within a study. For example, data on champion performance may be relevant also for reporting on implementation strategies employed and efforts to tailor strategies. Finally, champion impact measures, which are commonly measured determinants (i.e., organizational readiness for change and implementation climate) and implementation outcomes from Proctor and colleagues (2011), could be used for other non-champion-related study aims.

A limitation of the proposed model is that the literature review upon which the model is based was not a systematic review. However, the model development was based upon a 2018 comprehensive review, recent studies published after the review were published, and foundational studies from other fields not included in the review. Another limitation is that the model has not yet been tested empirically. Implementation science has many conceptual models already (Nilsen, 2015; Tabak et al., 2012), so it is reasonable to question whether another is necessary. However, this model fills the gap of specifying proposed relationships between the many champion-related constructs that have been identified and described in the literature. The goal is to move beyond descriptive findings about champions, which suggest champions are important and that some champions may be better than others, to prospective studies of champion impact. The model does so by categorizing modifiable factors, identified in the literature, that influence a champion’s potential impact and activities champions can perform to affect specific types of implementation outcomes. Over time, I hope that application of the model in new prospective studies that test the proposed relationships will lead to improvements to the model, as needed, and enhance its utility for future research and for practitioners serving in a champion role.

## Supplementary Material

Supplemental Figure

## Figures and Tables

**Table 1.** Potential measures of model constructs.

Construct	Selected dimensions	Potential measures	Example of model application
			Primary care provider serving as a champion for medication-assisted treatment (MAT) in her clinic
Champion perceptions of the innovation	Relative advantage Compatibility Complexity	Kegler et al. (2018) Huijg et al. (2014)	Survey the champion on perceptions of the relative advantage, compatibility, and complexity of MAT
Champion Commitment	Willingness to allocate time Willingness to put reputation at stake	Howell et al. (2005) Holt et al. (2007)	Survey the champion about her level of commitment to the champion role (e.g., willingness to allocate time to champion activities).
Champion experience and self-efficacy	Experience and self-efficacy with the innovation Experience and self-efficacy with leading organizational change	Huijg et al. (2014) Tucker et al. (2009)	Survey the champion on her knowledge, experience, and self-efficacy related to the innovation and leading organizational change
Organizational support for the champion	Available resources Leadership support	Walker et al. (2019) Helfrich et al. (2009)	Survey of the champion about her perceptions about support she is receiving from her clinic leadership for performing the champion role (e.g., reward/recognition for the role, necessary resources, dedicated time)
Champion performance	*Pre-implementation* Communicating the need for and benefits of the innovation Convening stakeholders Securing needed resources *Implementation* Facilitate users’ development of knowledge and skills Troubleshoot problems Monitor use of the innovation	Standardized reporting template provided to the champion to document activities, such as Resources allocated to the implementation effort Number of consultations held with peers to answer questions Problems identified by users and actions taken	In the pre-implementation phase, the champion uses the standardized reporting template to document actions taken for communicating the need for and benefits of MAT, stakeholder meetings, and resources secured for the implementation effort
Peer perceptions of the champion	*Pre-implementation* Expresses enthusiasm and confidence Persists under adversity Gets the right people involved Relative advantage Compatibility Complexity *Implementation* Coordinating activities Troubleshooting problems	Howell et al. (2005) Kegler et al. (2018) Huijg et al. (2014) Aarons et al. (2014) Helfrich et al. (2009) Ehrhart et al. (2015)	In the pre-implementation phase, clinicians who are intended users of MAT in the clinic complete a survey about the champion’s enthusiasm, persistence, and ability to engage the right people in the implementation. The survey also includes items about their own perceptions about the relative advantage, compatibility, and complexity of MAT
Champion impact	*Pre-implementation* Acceptability Appropriateness Feasibility Organizational readiness for change *Implementation* Implementation climate Adoption Penetration Fidelity Implementation costs	Weiner et al. (2017) Holt et al. (2007) Shea et al. (2014) Jacobs et al. (2014)	In the pre-implementation phase, intended users of MAT respond to a survey about the acceptability, appropriateness, and feasibility of MAT as well as their clinic's readiness for implementing MAT
