# Supplementary figures and images for: A conceptual model to guide research on the activities and effects of innovation champions
Source: Implement Res Pract. 2021 Mar 23;2:2633489521990443. doi: 10.1177/2633489521990443 (PMC8445003; doi:10.1177/2633489521990443)

**Figure 2: A Conceptual Model of Champion Impact with Additional Dimensions**

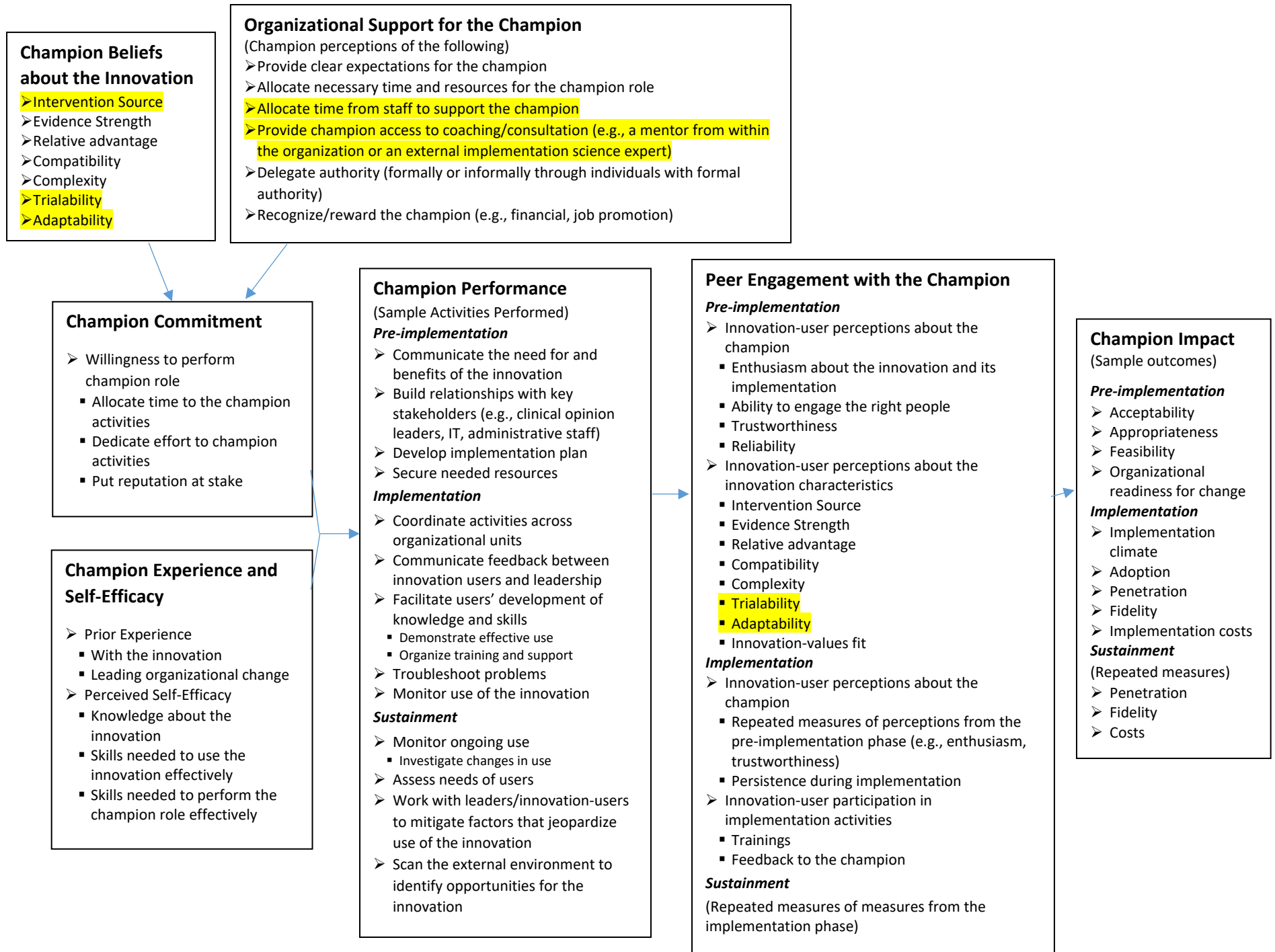

Supplement: sj-pdf-1-irp-10.1177_2633489521990443 – Supplemental material for A conceptual model to guide research on the activities and effects of innovation champions [file sj-pdf-1-irp-10.1177_2633489521990443.pdf]
