# Supplementary material for: A conceptual model to guide research on the activities and effects of innovation champions
Source: Implement Res Pract. Author manuscript; Available in PMC 2021 Sep 16. (PMC8445003; doi:10.1177/2633489521990443)
Supplement: Supplementary file 1 [file NIHMS1733166-supplement-Supplemental_Figure.pdf]

Figure 2: *A Conceptual Model of Champion Impact with Additional Dimensions*

Champion Beliefs about the Innovation

- Intervention Source
- Evidence Strength
- Relative advantage
- Compatibility
- Complexity
- Trialability
- Adaptability

Organizational Support for the Champion

(Champion perceptions of the following)

- Provide clear expectations for the champion
- Allocate necessary time and resources for the champion role
- Allocate time from staff to support the champion
- Provide champion access to coaching/consultation (e.g., a mentor from within the organization or an external implementation science expert)
- Delegate authority (formally or informally through individuals with formal authority)
- Recognize/reward the champion (e.g., financial, job promotion)

Champion Commitment

- Willingness to perform champion role
  - Allocate time to the champion activities
  - Dedicate effort to champion activities
  - Put reputation at stake

Champion Experience and Self-Efficacy

- Prior Experience
  - With the innovation
  - Leading organizational change
- Perceived Self-Efficacy
  - Knowledge about the innovation
  - Skills needed to use the innovation effectively
  - Skills needed to perform the champion role effectively

Champion Performance

(Sample Activities Performed)

Pre-implementation

- Communicate the need for and benefits of the innovation
- Build relationships with key stakeholders (e.g., clinical opinion leaders, IT, administrative staff)
- Develop implementation plan
- Secure needed resources

Implementation

- Coordinate activities across organizational units
- Communicate feedback between innovation users and leadership
- Facilitate users' development of knowledge and skills
  - Demonstrate effective use
  - Organize training and support
- Troubleshoot problems
- Monitor use of the innovation

Sustainment

- Monitor ongoing use
  - Investigate changes in use
- Assess needs of users
- Work with leaders/innovation-users to mitigate factors that jeopardize use of the innovation
- Scan the external environment to identify opportunities for the innovation

Peer Engagement with the Champion

Pre-implementation

- Innovation-user perceptions about the champion
  - Enthusiasm about the innovation and its implementation
  - Ability to engage the right people
  - Trustworthiness
  - Reliability
- Innovation-user perceptions about the innovation characteristics
  - Intervention Source
  - Evidence Strength
  - Relative advantage
  - Compatibility
  - Complexity
  - Trialability
  - Adaptability
  - Innovation-values fit

Implementation

- Innovation-user perceptions about the champion
  - Repeated measures of perceptions from the pre-implementation phase (e.g., enthusiasm, trustworthiness)
  - Persistence during implementation
- Innovation-user participation in implementation activities
  - Trainings
  - Feedback to the champion

Sustainment

(Repeated measures of measures from the implementation phase)

Champion Impact

(Sample outcomes)

Pre-implementation

- Acceptability
- Appropriateness
- Feasibility
- Organizational readiness for change

Implementation

- Implementation climate
- Adoption
- Penetration
- Fidelity
- Implementation costs

Sustainment

(Repeated measures)

- Penetration
- Fidelity
- Costs
